# Environmental temperature and exercise modality independently impact central and muscle fatigue among people with multiple sclerosis

**DOI:** 10.1177/2055217317747625

**Published:** 2017-12-21

**Authors:** Geetika Grover, Michelle Ploughman, Devin T Philpott, Liam P Kelly, Augustine J Devasahayam, Katie Wadden, Kevin E Power, Duane C Button

**Affiliations:** School of Human Kinetics and Recreation, Memorial University, Canada; Recovery and Performance Laboratory, Memorial University, Canada; School of Human Kinetics and Recreation, Memorial University, Canada; Recovery and Performance Laboratory, Memorial University, Canada; Recovery and Performance Laboratory, Memorial University, Canada; School of Human Kinetics and Recreation, Memorial University, Canada; BioMedical Sciences, Memorial University, Canada

**Keywords:** Muscle fatigue, aerobic exercise, electromyography, triceps surae, plantar flexor, heat sensitivity

## Abstract

**Background:**

Heat sensitivity and fatigue limit the ability of multiple sclerosis patients to participate in exercise.

**Objective:**

The purpose of this study was to determine the optimal aerobic exercise parameters (environmental temperature and exercise modality) to limit exercise-induced central and muscle fatigue among people with multiple sclerosis.

**Methods:**

Fourteen people with multiple sclerosis with varying levels of disability completed four randomized exercise sessions at 65% of the maximal volume of oxygen: body-weight supported treadmill cool (16°C), body-weight supported treadmill room (21°C), total-body recumbent stepper cool and total-body recumbent stepper room. Maximum voluntary contraction, electromyography, and evoked contractile properties were collected from the more affected plantar flexors along with subjective levels of fatigue, body temperature and perceived level of exertion.

**Results:**

Exercise in cooler room temperature increased maximum voluntary contraction force (*p* = 0.010) and stabilized body temperature (*p* = 0.011) compared to standard room temperature. People with multiple sclerosis experienced greater peak twitch torque (*p* = 0.047), shorter time to peak twitch (*p* = 0.035) and a longer half relaxation time (*p* = 0.046) after total-body recumbent stepper suggestive of less muscle fatigue.

**Conclusion:**

Cooling the exercise environment limits the negative effects of central fatigue during aerobic exercise and using total-body recumbent stepper (work distributed among four limbs) rather than body-weight supported treadmill lessens muscular fatigue. Therapists can titrate these two variables to help people with multiple sclerosis achieve sufficient exercise workloads.

## Introduction

Exercise training improves muscular strength, aerobic capacity, walking ability and health-related quality of life among people with multiple sclerosis (PwMS).^1^ However, due to disability, fatigue and heat sensitivity, most PwMS do not exercise^2,3^ and so they do not obtain its potential benefits. Aerobic fitness, in particular, is associated with less brain atrophy and better cognitive function in multiple sclerosis (MS)^4^ but at the same time, aerobic exercise increases body temperature which many MS patients find intolerable.^5^ For example, after leg cycling, MS participants experienced greater increases in body temperature compared to controls which was correlated with prolonged perceived leg fatigue.^6^ There is a need to find innovative and tailored exercise protocols so that PwMS can achieve optimal exercise workloads.^7^

Exercise-induced fatigue could be due to impaired motor drive from the central nervous system (CNS) (central fatigue) or reduced capacity from within the muscle.^8,9^ Research supports the interplay of both mechanisms. Demyelination of motor pathways,^10^ impaired thermoregulation, and transient increase in symptoms with passive heat exposure^5^ point to a central origin. However, decreased muscle peak twitch (PT) force output and prolonged half-relaxation time (HRT) during fatiguing electrical stimulation of the tibialis anterior in PwMS^11^ is suggestive of peripherally induced fatigue within the muscle.^11,12^

Aerobic exercise is typically performed on a treadmill but can be adapted for PwMS who have balance difficulties by using seated or recumbent methods. For example, Pilutti et al.^13^ compared the safety and tolerability of the total body recumbent stepper (TBRS) to the body-weight supported treadmill (BWST) and found that 12 weeks of TBRS and BWST training reduced perceived fatigue, although PwMS reported a more positive experience with the TBRS.^13^ It is conceivable that distributing the workload between four limbs rather than two could lessen excessive lower limb fatigue. Choosing the appropriate exercise modality (seated or upright) and cooling the temperature of the exercise environment are simple methods that therapists and patients can employ in order to titrate aerobic exercise parameters to make training achievable.

The primary aim of this study was to determine the acute effects of combining temperature ('cool', 16°C or 'room', 21°C) and exercise modality (BWST or TBRS) on lower extremity central fatigue (maximum voluntary contraction (MVC)), muscle fatigue (evoked contractile properties), body temperature, and perceived levels of fatigue. We hypothesized that: (a) exercising in a cooler temperature would prevent post-exercise decreases in MVC and electromyography (EMG) and (b) due to distributed workload between all four limbs, PwMS would have less perceived fatigue following the TBRS compared with the BWST. In order to understand potential mechanisms, our exploratory aims were to evaluate the relationships between exercise-induced changes in body temperature, subjective fatigue, the extent of clinical disability, and changes in neuromuscular performance.

## Methods

Following approval of the Health Research Ethics Board (ref. no. 14.102), participants were recruited from outpatient rehabilitation services and the local MS clinic. From these participants, 14 PwMS (10 females) were included; they all met the following inclusion criteria: (a) diagnosis using the McDonald criteria;^14^ (b) a negative Physical Activity Readiness Questionnaire (PAR-Q)^15^ screening; (c) being relapse-free during the past three months; (d) taking no medication that affects heart response to exercise; (e) having no musculoskeletal impediment to exercise; and (f) scoring greater than 24 on the Montreal Cognitive Assessment (MoCA).^16^

### Experimental design

During the first session, demographic and MS-related data were collected (age, years since diagnosis, Fatigue Impact Scale (FIS)).^17^ PwMS self-reported their degree of heat sensitivity using a visual analogue scale (VAS); zero being not at all sensitive and 100 being extremely sensitive. Lower limb strength was tested using manual muscle testing, spasticity using the Modified Ashworth Scale and self-selected walking velocity using an instrumented walkway (Protokinetics, Havertown, Pennsylvania, USA).

**Figure 1.**
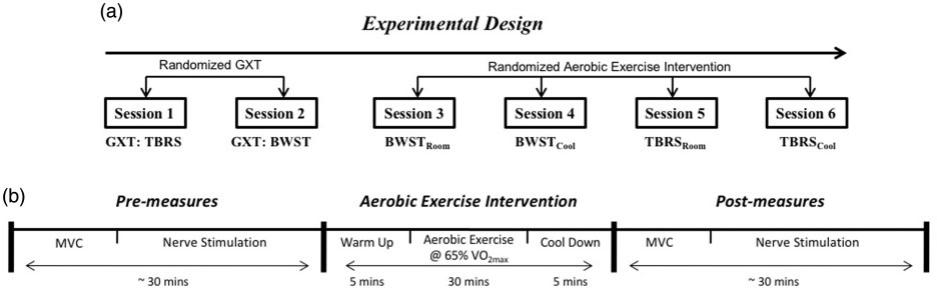
(a) Experimental design. Graded maximal exercise tests (GXTs) were performed one week apart. Sessions 3–6 were performed in randomized order one week apart; body-weight support treadmill at room 21°C (BWST_room_); BWST at 16°C (BWST_cool_); total-body recumbent stepper at 21°C (TBRS_room_); TBRS at 16°C (TBRS_cool_). (b) Intervention session protocol: before and after exercise, participants performed two maximal voluntary contractions (MVCs) and received posterior tibial nerve electrical stimulation to measure plantar flexor twitch contractile properties from lateral gastrocnemius (LG) and soleus (SOL) muscles. VO_2max_: maximal volume of oxygen.

BWST and TBRS graded maximal exercise tests (GXTs) were performed (Figure 1(a)) and maximal volume of oxygen (V̇O_2max_) was used to assign 60–65% of heart rate (HR) reserve for 30 min of aerobic exercise (including a five-minute warm-up and cool down). The participants completed four randomized exercise sessions, one week apart either on TBRS or BWST in a temperature controlled room at 16°C (cool) or 21°C (room); BWST_cool_, BWST_room_, TBRS_cool_, TBRS_room_ (Figure 1(a)). Rate of perceived exertion (RPE; Borg Scale (10-point)) and HR were recorded at intervals 1, 15, and 30 min during exercise. Prior to each exercise session, participants were prepared for EMG (Figure 1(b)) and performed isometric contractions of the plantar flexors at various low intensities as a warm-up. Before and after exercise, participants performed two MVCs, received posterior tibial nerve stimulation, rated their perceived fatigue on a 100 mm VAS, and had body temperature recorded using a tympanic thermometer (Thermoscan, Braun, Kronberg, Germany).

### Maximal exercise tests

V̇O_2max_ test was performed on both the BWST using 10% body weight support (Sport Art T625M/T52 MD-Rehabilitation Commercial Treadmill, USA) and the TBRS (NuSTEP T4r Recumbent stepper, Michigan, USA), according to guidelines adapted from stroke best practices.^18^ Height, weight, age, resting HR, and blood pressure were taken prior to the GXT. The metabolic cart (Moxus Metabolic Systems, AEI Technologies, Inc., Pittsburgh, Pennsylvania, USA) was calibrated and expired air was analyzed breath-by-breath using a mask (HR: Polar V800, Polar Electro Oy, Professorintie 5, FI-90440 Kempele, Finland).

### Measurement of plantar flexor muscle force and electromyography

Participants sat with their weaker leg flexed (i.e. 90° at the hip, knee, and ankle joints) and foot mounted in a modified boot apparatus equipped with a load cell, and performed two five-second MVCs. Participants were told to push into plantar flexion as hard as possible for five seconds. Force was sampled at 1 kHz, amplified (×1000) and averaged. EMG was recorded via surface 10 mm electrodes (MediTrace Pellet Ag/AgCl, Graphic Controls Ltd., Buffalo, New York, USA) placed longitudinally 2 cm apart over the lateral gastrocnemius (LG) and soleus (SOL) muscles of the weaker leg (determined by manual muscle test) and a ground electrode was secured on the lateral epicondyle of the femur. Signals were amplified (1000×), filtered using a Butterworth filter with a pass-band of 10–500 Hz and analog-digitally converted at a sampling rate of 1000 Hz (Biopac MP150WSW, Biopac Systems Inc., Holliston, Mississippi, USA). Data were recorded and analyzed (Acknowledge 4.1, Biopac Systems Inc.). MVC forces were measured as the peak force recorded during each contraction. For the LG and SOL muscles, root mean square (RMS) EMG was calculated over 500 ms about the peak force amplitude during the MVC for each muscle.

### Assessment of evoked contractile properties

To assess twitch contractile properties in LG and SOL muscles; electrical stimulation was applied to the posterior tibial nerve via Ag/AgCl electrodes placed in the popliteal fossa (cathode) and over the tibial tuberosity (anode). Current pulses (200 µs duration, 100–400 mA) were delivered via a constant current stimulator (DS7AH; Digitimer, Welwyn Garden City, Hertfordshire, UK). Stimulation intensity was increased until PT torque of LG and SOL plateaued. Evoked contractile properties included: (a) PT torque, (b) time to peak twitch (TPT), and (c) HRT – the time it took for the PT torque to reduce to half of its maximum amplitude. Lower PT and longer TPT are indicative of fatigue and lower efficiency of fiber cross-bridge cycling, respectively.^19^

### Statistical analysis

To determine whether the exercise conditions were at the equivalent aerobic intensity, repeated measures analysis of variance (ANOVA) for exercise condition (TBRS, BWST) and temperature (cool, room) were performed on HR, power output, and RPE averaged across three time points (1 min, 15 min, 30 min) during the 30 min of aerobic exercise. A repeated measures ANOVA for exercise condition (TBRS, BWST) by temperature (cool, room) was also performed on change scores (CSs) (post minus pre; MVC_cs_, RMS EMG_cs_, PT_cs_, TPT_cs_, HRT_cs_, fatigue_cs_, body temp_cs_,) and on perceived level of fatigue.

Based on evidence that body temperature relates to changes in central drive in PwMS following exercise,^20^ correlations were performed between post-exercise MVC torque, degree of heat sensitivity and change in body temperature for each exercise modality (BWST; TBRS) in the room temperature condition. In exploratory analyses of MS biomarkers, the relationship between disability (Expanded Disability Status Scale (EDSS)) and variables at baseline (i.e. the average of pre-MVC, RMS EMG, PT, TPT, HRT, and perceived fatigue) was tested using Pearson correlations (Pearsons’s *r*: *p* < 0.05, uncorrected).

If significant main interactions were found (*F*-ratio: *p* < 0.05), a Bonferroni post-hoc test was performed. If the assumption of sphericity was violated, the corrected value for non-sphericity with Greenhouse-Geisser epsilon was reported. Effect sizes were reported as partial eta-squared (*n*_p_^2^) where 0.02 is considered a small effect, 0.13 moderate and more than 0.26, a large effect.^21^ Data are reported as means and standard errors (SEs) (SPSS 19.0, IBM Corporation, Armonk, New York, USA).

## Results

**Figure 2.**
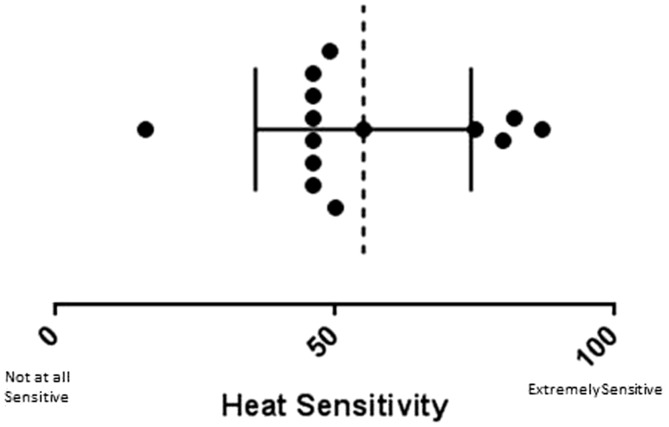
Ratings of heat sensitivity rating on heat sensitivity visual analog scale (VAS; score out of 100). Each circle is a participant. Dashed line is the mean and horizontal error bar is the standard deviation (SD).

Three of the 14 individuals were unable to complete the BWST protocol, and therefore were excluded from repeated measures statistical analyses (*n* = 11). All participants reported heat sensitivity (Figure 2) and 11 had lower extremity weakness (Table 1). Participant characteristics are reported in Table 1 and raw data for voluntary and evoked contractile properties are presented in Table 2.

**Table 1.** Participants’ clinical characteristics.

Participant	EDSS	Age	Sex	Type of MS	FIS	LE summed strength	LE spasticity	Use of gait aid	Walking speed cm/s
1	2.0	31	Male	RRMS	16	80	–	–	128.19
2	1.5	38	Male	RRMS	41	61	–	+	79.15
3	4	56	Female	RRMS	20	74	–	–	97.29
4	3.5	52	Male	RRMS	49	61	–	–	84.37
5	1.5	65	Female	RRMS	3	74	–	+	110.86
6	7	55	Female	SPMS	41	47	+	+	32.16
7	1.5	43	Female	RRMS	12	80	–	–	119.75
8	6.5	71	Female	SPMS	30	50	+	+	16.45
9	1	28	Female	RRMS	2	78	–	–	123.54
10	6.5	59	Female	PPMS	26	57	–	+	46.06
11	0	29	Female	RRMS	0	80	–	–	76.50
12	2	60	Male	RRMS	41	80	–	–	119.22
13	0	53	Female	RRMS	31	61	–	–	143.20
14	1	50	Female	RRMS	36	66	–	–	124.13

EDSS: Expanded Disability Status Scale; FIS: Fatigue Impact Scale; LE: lower extremity; MS: multiple sclerosis; PPMS: primary progressive MS; RRMS: relapsing–remitting MS; SPMS: secondary progressive MS.

Strength/80 summed manual muscle tests of major muscles in both lower limbs.

Present (+), absent (–); walking speed, self-selected pace (cm/s).

**Table 2.** Raw data for voluntary and evoked contractile properties.

Measure	Exercise	Temp	Pre- (*n* = 11)	Post- (*n* = 11)
**MVC** *(Nm)*	BWST	Cool	89.81 (± 62.41)	100.3 (± 66.83)
**MVC** *(Nm)*	TBRS	Cool	95.18 (± 53.19)	108.9 (± 61.89)
**MVC** *(Nm)*	BWST	Room	112.3 (± 68.66)	101.1 (± 55.72)
**MVC** *(Nm)*	TBRS	Room	98.90 (± 62.72)	93.68 (± 64.79)
**LG EMG** *(RMS)*	BWST	Cool	0.063 (± 0.0359)	0.065 (± 0.0402)
**LG EMG** *(RMS)*	TBRS	Cool	0.062 (± 0.0334)	0.072 (± 0.0488)
**LG EMG** *(RMS)*	BWST	Room	0.076 (± 0.0446)	0.062 (± 0.0404)
**LG EMG** *(RMS)*	TBRS	Room	0.082 (± 0.0582)	0.072 (± 0.0593)
**HRT** *(ms)*	BWST	Cool	121.2 (±25.96)	110.3 (±21.22)
**HRT** *(ms)*	TBRS	Cool	117.9 (±22.37)	104.6 (±27.80)
**HRT** *(ms)*	BWST	Room	132.8 (±34.50)	108.1 (±28.23)
**HRT** *(ms)*	TBRS	Room	107.4 (±26.94)	108.9 (±24.20)
**PT** *(Nm)*	BWST	Cool	15.09 (± 9.644)	13.76 (± 7.101)
**PT** *(Nm)*	TBRS	Cool	14.09 (± 6.245)	16.76 (± 10.12)
**PT** *(Nm)*	BWST	Room	15.07 (± 6.702)	14.99 (± 8.750)
**PT** *(Nm)*	TBRS	Room	14.46 (± 6.835)	16.75 (± 9.719)
**TPT** *(ms)*	BWST	Cool	0.15 (± 0.0213)	0.14 (± 0.0196)
**TPT** *(ms)*	TBRS	Cool	0.16 (± 0.0255)	0.15 (± 0.0347)
**TPT** *(ms)*	BWST	Room	0.13 (± 0.0504)	0.14 (± 0.0255)
**TPT** *(ms)*	TBRS	Room	0.17 (± 0.0229)	0.14 (± 0.0274)

Maximal Voluntary Contraction (MVC); Lateral Gastrocnemius (LG); Electromyography (EMG); Newton Meter (Nm); Root Mean Square (RMS); Temperature (Temp); Half Relaxation Time (HRT); Peak Twitch (PT); Time to Peak Twitch (TPT); milliseconds (ms); Total body recumbent stepper (TBRS); Body-weight supported treadmill (BWST).

### The intensity of exercise was equivalent between exercise modalities

There was no significant main effect of environmental temperature or exercise modality on HR_avg_ or power output_avg,_ (data not shown). Although there was no significant main effect of environmental temperature on RPE_avg_ (*F*_(1,11)_ = 1.00, *p* = 0.34, *n*_p_^2^ = 0.091), there was a significant main effect of exercise modality, (*F*_(1,11)_ = 8.07, *p* = 0.018, *n*_p_^2^ = 0.447) with a higher level of perceived exertion during TBRS (4.54 ± 0.278) compared to BWST (3.97 ± 0.293). There was no significant interaction of temperature and exercise modality (*p* = 0.17).

### Maximal plantar flexor activation was temperature-dependent

**Figure 3.**
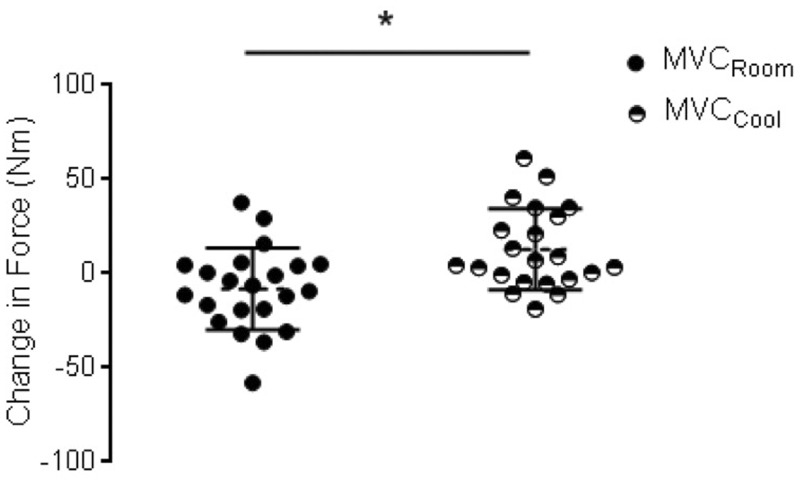
Temperature effects on maximal voluntary contraction (MVC). Change in MVC torque in room (black) and cool (black/white) collapsed across exercise modalities. *Indicates a significant (*p*<0.05) main effect of temperature condition.

There was a significant main effect of environmental temperature on MVC_cs_ (Figure 3; *F*_(1,11)_ = 9.86, *p* = 0.010, *n*_p_^2^ = 0.497). MVC torque increased post-exercise in cool temperature (12.13 ± 4.54 Nm) and decreased in room temperature (–8.20 ± 5.13 Nm). There was a near main effect of temperature (*F*_(1,11)_ = 6.73, *p* = 0.057, *n*_p_^2^ = 0.254) but no effect of exercise modality on LG EMG_cs_. LG EMG remained the same post-exercise in cool temperature (0.002 ± 0.003 mV) but decreased in room temperature (–0.01 ± 0.004 mV).

There was no effect of exercise modality on MVC_cs,_ LG EMG or SOL EMG and no significant interaction between temperature and exercise modality. There was also no effect of temperature on SOL EMG_CS_ (data not shown).

### Exercise modality affected evoked contractile properties

Following BWST there was evidence of plantar flexor fatigue. There was a significant main effect of exercise modality on plantar flexor PT_cs_, (Figure 4; *F*_(1,11)_ = 5.11, *p* = 0.047, *n*_p_^2^ = 0.338) with decreased PT amplitude following BWST (–0.71 ± 0.982 Nm) and an increase following TBRS (2.48 ± 1.173 Nm). There was also a significant main effect of exercise modality on TPT_cs_ (*F*_(1,11)_ = 5.92, *p* = 0.035, *n*_p_^2^ = 0.372) with prolonged TPT following BWST (0.004 ± 0.009 ms) and shortened TPT following TBRS (–0.019 ± 0.007 ms). Further, there was a significant main effect of exercise modality on HRT_cs,_ (*F*_(1,11)_ = 5.21, *p* = 0.046, *n*_p_^2^ = 0.343) with shortened HRT following exercise on a BWST (–17.80 ± 2.845 ms) compared to exercise on TBRS (–5.89 ± 4.487 ms).

**Figure:**
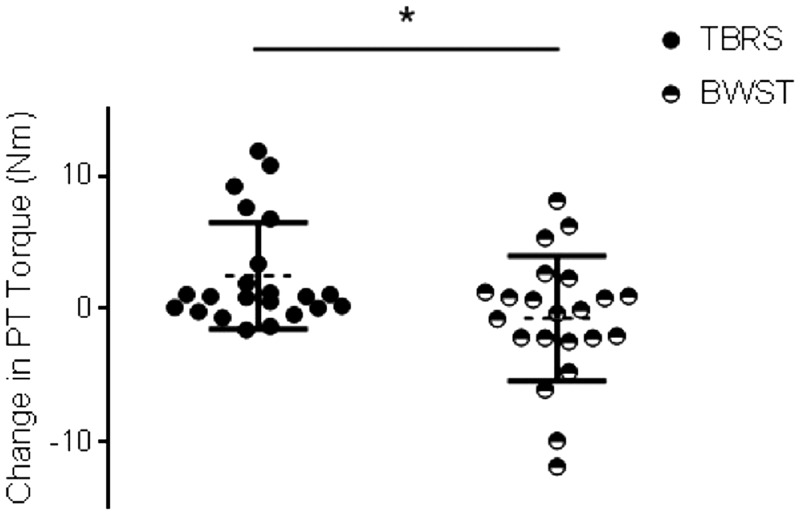
**Figure 4.** Exercise modality effects on evoked contractile properties. Change in peak twitch (PT) in body-weight support treadmill (BWST) (black/white circle) and total-body recumbent stepper (TBRS) (black circle) collapsed across temperature conditions. *Indicates a significant (*p*<0.05) main effect of exercise modality.

There was no effect of temperature on PT_cs,_ TPT_cs_ or HRT_cs_, and no significant interaction between temperature and exercise modality (data not shown).

### Effects of exercise modality on perceived level of fatigue and body temperature

There were significant main effects of exercise modality and environmental temperature on body temp_cs_ (Table 3; *F*_(1,9)_ = 7.61, *p* = 0.022, *n*_p_^2^ = 0.458, *F*_(1,9)_ = 10.08, *p* = 0.011, *n*_p_^2^ = 0.528, respectively). Greater increases in body temperature were observed following exercise in room temperature (0.33 ± 0.104°C) compared to cool temperature (–0.15 ± 0.123°C). There was a greater increase in body temperature following TBRS (0.29 ± 0.094°C) compared to BWST (0.025 ± 0.126°C). There was no significant main effect of exercise modality or environmental temperature on perceived level of fatigue_cs_ and no significant interaction of exercise modality and temperature (data not shown).

**Table 3.** Raw data for change in body temperature (data are reported as mean (M) ± standard deviation (SD))

Measure	Exercise	Temp	Pre-(*n* = 11)	Post-(*n* = 11)
Body temp (°C)	BWST	Cool	36.65 (±0.454)	36.45 (±0.780)
Body temp (°C)	TBRS	Cool	36.65 (±0.418)	36.74 (±0.559)
Body temp (°C)	BWST	Room	36.49 (±0.287)	36.73 (±0.402)
Body temp (°C)	TBRS	Room	36.35 (±0.462	36.78 (±0.524)

BWST: Body-weight supported treadmill; TBRS: Total body recumbent stepper; Temp: temperature.

### Relationship between body temperature, degree of heat sensitivity and neuromuscular performance

Since there was little change in body temperature in the cool condition, changes in body temperature in the room temperature conditions were compared to the corresponding post-exercise MVC torque. Higher body temperature was correlated with declines in post-exercise MVC torque in BWST (Figure 5; *r* = 0.65, *p* = 0.028) and a nearly significant negative relationship in TBRS (*r* = 0.58, *p* = 0.075). There was no relationship between body temperature and evoked contractile properties. Nether was there a relationship between perceived fatigue, degree of heat sensitivity, MVC, EMG, or evoked contractile properties.

**Figure:**
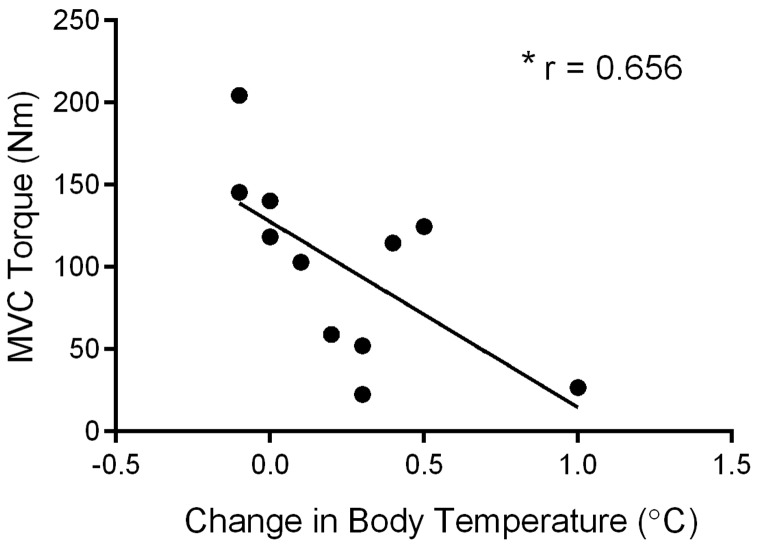
**Figure 5.** Relationship between body temperature and post-exercise maximal voluntary contraction **(**MVC). Following body-weight support treadmill at room 21°C (BWST_room_)_,_****lower MVC torque was significantly correlated with increases in body temperature. *Indicates a significant (*p* < 0.05) correlation.

### Relationship between MS-related disability and neuromuscular performance

As expected, higher EDSS scores were significantly correlated with lower MVC (*r* = –0.54, *p* = 0.045), lower LG (*r* = 0.62, *p* = 0.022), SOL RMS EMG (*r* = –0.66, *p* = 0.013), and greater baseline perceived fatigue (*r* = 0.67, *p* = 0.012).

## Discussion

The main findings of the current study were (a) with large effect sizes, a cooler environment (16°C) limited elevation of body temperature and increased plantar flexor MVC torque and protected against decrements in EMG after exercise, suggesting an effect of temperature on CNS drive and (b) exercising on a recumbent stepper using all four limbs enhanced plantar flexor contractile properties as compared to treadmill training, suggesting an effect of exercise modality on the excitation-contraction coupling of the muscle. These findings occurred despite the fact that the exercise interventions were matched for workload and participants perceived the exercise on the TBRS as *more* strenuous than the BWST. Finally, the neuromuscular performance measures, specifically MVC and RMS EMG of the lower limb, correlated with clinical disability (EDSS), strengthening their usefulness as biomarkers of subtle MS-related sensorimotor change.

### Environmental temperature and central drive

In the present study, exercising in room temperature (raising body temperature) led to decreased MVC torque and LG EMG, with no concomitant changes in evoked contractile properties post-exercise, pointing to CNS mechanisms underlying these changes. Other studies support such a theory. When PwMS were exposed to passive body heating, walking, force generation capacity, and increased fatigue perception were affected.^22^ Furthermore, passively-heated PwMS had decreased corticospinal excitability as shown via increased resting motor threshold and decreased motor evoked amplitude.^22^ Increased body temperature may also alter action potential propagation in demyelinated or partially myelinated axons causing reduced conduction velocity and/or block.^5^ In the present study, exercising in cool temperature enhanced MVC torque with no change in LG EMG. The cool temperature may have alleviated heat-induced stress on the CNS, allowing for improved MVC performance.

Exercising in warm or cool environments, and rate of heat storage in the body, have been postulated to differentially activate an anticipatory response that regulates skeletal muscle power output and motor unit recruitment through afferent sensory inputs to the brain.^23^ Other studies have shown that, in PwMS, water immersion precooling before arm-leg ergometry exercise stabilized core temperature and improved 25-foot walk performance^24^ and that the use of cooling suits improved walking speed and lower-limb strength.^25^ Decreasing body heat storage, especially during exercise, may allow for improved CNS function and enhanced neuromuscular performance. Since comfort is likely important for exercise compliance, the longer-term effects (over hours and days) of temperature and fatigue on neuromuscular measures and functional tasks is an important area for future study.

### Exercise modality

Based on our findings that BWST resulted in lower PT force and longer TPT, the BWST may create impaired excitation-contraction coupling of the plantar flexor muscles, indicating greater muscle fatigue, compared to the TBRS. Conversely, the TBRS facilitated the excitation-contraction coupling of the plantar flexor muscles demonstrated by increased PT force and shorter TPT. The enhancement of plantar flexor muscle contractile properties following the TBRS may be related to exercise-induced post-activation potentiation (PAP); enhanced calcium kinetics, myosin phosphorylation, and reduced muscle stiffness after non-fatiguing contractions.^26^ Biomechanically, the distributed workload between the upper and lower limb muscles required to perform the TBRS at a comparable physiological workload as submaximal BWST exercise, that predominately engages muscles of the lower limb, may help to explain our findings. For those with fatigue and heat sensitivity, choosing the TBRS in a cooler environment may allow patients to reach optimal aerobic exercise training levels.

### Perceptions of fatigue

In a clinical setting, the patient’s subjective report of heat sensitivity and fatigue during exercise may be used when titrating aerobic training intensity. However, in this study, perceived fatigue following exercise was not related to objective measures of central or muscular fatigue, illustrating a disconnect between psychological vs physiological aspects of fatigue in PwMS following exercise. This disconnect suggests that subjective reports may not be sensitive enough to gauge heat-related fatigue. Dawes et al.^20^ showed that PwMS reporting general fatigue could train at comparable levels to those without fatigue suggesting that clinicians, in addition to obtaining subjective reports, should focus on objective markers of exercise intensity such as HR to gauge exercise parameters. Our findings suggest that, at least within a training session, the TBRS may be more tolerable especially for those with higher levels of MS-related disability. However, due to the importance of the exercise-induced stress response that is incurred from challenging exercise,^27^ transition to the BWST may be warranted as training progresses.

### Detecting subclinical changes in neuromuscular performance

We found not only that there was an inverse relationship between EDSS score and MVC force and EMG of the plantar flexor muscles, but also the neuromuscular measures detected subtle deficits sometimes unbeknownst to the participant. Others have demonstrated that MVC, central activation,^28^ and evoked contractile properties^10^ are decreased in PwMS compared to controls and there is a negative correlation between EDSS and measures of corticospinal excitability.^29^ In a disease where most of the degeneration is subclinical and where lesions can outnumber clinical relapses 10 to one,^30^ there are opportunities to incorporate neuromuscular performance outcomes as potential biomarkers of MS disease progression.

### Study limitations

This study makes important strides in developing tailored aerobic exercise interventions for PwMS. However, there were some limitations. Even though effect sizes were moderate to large, the sample size was small and because of inability to complete the BWST, participants with EDSS >6 were underrepresented.

Body temperature was measured using the tympanic method. The most sensitive methods to measure thermoregulation and changes in core temperature are invasive, and are recorded using rectal and esophageal techniques. However, the aim of the present study was to use methods that could be easily incorporated into clinical practice and inform best practice guidelines for clinical exercise prescription.

## Conclusions

Cooling the temperature of the training environment lessens central fatigue while choosing a modality that distributes workload among four limbs (TBRS) rather than two (BWST) limits muscular fatigue. Therapists can modify these parameters in order to achieve optimal aerobic exercise training levels among PwMS.
